# Sibling Separation Due to Parental Divorce: Diagnostic Aspects

**DOI:** 10.3390/ijerph19106232

**Published:** 2022-05-20

**Authors:** Aleksandra Lewandowska-Walter, Magdalena Błażek

**Affiliations:** 1 Institute of Psychology, University of Gdańsk, 80-309 Gdańsk, Poland; 2 Department of Psychology, Gdańsk Medical University, 80-210 Gdańsk, Poland; magdalena.blazek@gumed.edu.pl

**Keywords:** siblings’ relationships, sibling separation, divorce, parental conflict, diagnostic play

## Abstract

Separation of siblings is one of the most difficult diagnostic problems faced by psychologists. Such situations are happening more often in the face of the increasing number of divorces and breakdown of relationships. Therefore, a diagnostic task becomes an in-depth assessment of intra-family relationships, ties connecting family members, the preferences of individual people and predicting the long-term consequences of the proposed solutions. The article is dedicated to this problem, and the issue is addressed through the theoretical perspective and the analysis of two cases, i.e., the situation of separated siblings. In the study of children, we present a relatively new method, based on the authors’ clinical experience, which could be used to diagnose the family situation of children. The first goal was to analyze the reasons for the separation of siblings whose parents were in conflict during the separation (first case study) and after the separation (second case study), as well as to assess the functioning of the children resulting from the family breakdown, and the decision to separate them from siblings. The analysis allowed identifying the areas of sibling functioning, which should become the subject of diagnosis when working on expert opinions in divorce cases, or cases establishing contact between parents and children. The second aim of the report was to assess the effectiveness of using play as a diagnostic method in a situation that is a source of stress for the child (family breakdown) and causes tension (the diagnostic process in which this topic is discussed).

## 1. Introduction

Parental divorce is a crisis situation that provides context for changes in family life [1,2]. In 55 years, the divorce rate has more than doubled in Europe: from 0.8 per 1000 people in 1964 to 1.8 in 2019. In 2019, the divorce rate in Poland amounted to 1.3 per 1000 people. It is a decrease of 0.4 as compared to the previous year and an increase of 0.3 as compared to 1995 divorce cases. Although Poland remains a country with one of the lowest rates of marital breakdown compared to other European countries, experts believe that this is still quite a high score. Moreover, in the coming years, we should expect increases, especially since there was a pandemic stagnation in the first half of last year [3]. In 2008, in Poland, the total number of divorces in families with children was 43,173 [4]; while in 2015, more than 67,000 spouses split up—39,100 of them were raising underaged children, more than a half of whom were 7–15 years old at the time of divorce [5]. In Poland, the issues of parental responsibility are determined by the Family and Guardianship Code (1964, as amended), which is the primary legal document in matters concerning parents’ obligations towards children.

According to Art. 95 of this Code, parental authority covers mainly the obligations and rights of the parents to exercise custody over the child and the child’s property, as well as the right to bring up the child; which means, among other things, that the child should obey their parents, who in turn have a duty to care for the child’s welfare and social interest. According to Art. 96 of the Family and Guardianship Code, parents are obliged to take care of the child’s physical and spiritual development and to prepare the child to work for the benefit of society, according to the child’s abilities. Such a provision means, in particular, the necessity to meet all (physical and psychological) needs of the child, understanding and recognizing their abilities and providing optimal conditions for the development of their abilities. In families who are not disturbed by symptoms of pathology or a conflict that prevents agreement, the parents make the decisions concerning the child together. However, the court intervenes when it is impossible for the parents to reach an agreement or when the care and education conditions constitute a threat to the proper functioning and development of the child.

In the psychological literature [6,7,8,9,10] concerning divorce and the impact of its dynamics on the well-being and psychological functioning of children, the considerations are linked to two related problems. The first is the short- and long-term consequences of parental conflict on the child. The second is the creation of guidelines for adequate childcare after the divorce, taking into account conflict as an important variable.

Studies on the effects of divorce on sibling relationships show a big variation in how children, as a family subsystem, cope with changes in the family [11]. It is emphasized that relations with siblings are among those few factors in children’s lives during the divorce and shortly after the divorce and complement the relations with parents [12]. The consequences of divorce stem not only from the separation of the child from the parent but also from the family malfunctioning before the divorce and/or difficult living conditions and conflicts after the divorce (e.g., on the level of parental cooperation) [13,14]. Children may experience conflict between their parents long before the parental decision to file for divorce. In the case of an open conflict, the child often acts as an intermediary between the arguing adults; while the consequences of the so-called silent conflict [15] may be more severe for the child because they obtain contradictory information from parents and they are uncertain as to what exactly are the concerns at the core of the conflict. (Are they related to the matters of the adults or children?)

Błażek et al. [16] showed that in cases of families examined by the court to assess the parenting capacity, the bond in the parent–child dyads was not forming properly, and moreover, the relationship between the child and one parent depended on the attitude of the other parent. In families with two or more children, divorce has a smaller impact on sibling relationships when parental conflict is less severe and significantly affects the quality of these relationships in high-conflict families [17]. In addition, high conflict divorce changes sibling relationships over the long term. Finally, as adults, siblings who grew up in divorced families have more conflicted relationships than siblings growing up in non-divorced families [17].

As a result of the parental conflict during divorce, the decisions made by them are not always in the best interest of the children but constitute a beneficial solution from the parents’ perspective [18]. According to Art. 107 of the Family and Guardianship Code, “Siblings should be raised together, unless the best interests of the child require a different decision”. The UN Convention on the Rights of the Child (UNCRC) also emphasizes the importance of sibling relations for the child’s well-being [19]. Therefore, separation of siblings after divorce is permissible, but only if it is in their best interest. The children’s right to be brought up with their siblings is particularly protected, and the parents should not, under any circumstances, seek to separate the siblings unless their best interest requires otherwise.

Statistical data show that in Poland, this kind of split custody is not common. In the divorce decree, minor siblings from the parents’ marriage were separated, entrusting the direct custody of some children to the mother and others to the father, but quite rarely—e.g., in 283 cases in 2014 and 250 cases in 2016 [20]. However, a similar phenomenon, the scale of which is not established by statistics, occurs when the relationships of cohabitating adults break up. However, when parents cannot agree on who should have primary custody or if they choose not to have shared custody, they can choose a solution whereby each adult has one child in their primary custody (in the case of 2-child families), or some of the children (in the case of larger families). In this case, the siblings are separated after the divorce and, as such, deal with another major change in their lives and lose one more resource—the bond with their brother or sister. When faced with a conflict, parents try to make sure that the siblings have contact with each other or that the child also meets the parent who is not their primary guardian. By presenting case studies of such families and analyzing the situation of siblings who grow up in them, we would like to draw attention to this issue and consider the change in the quality of life of siblings separated as a result of their parents’ divorce.

## 2. Theoretical Background

The functioning of siblings after their parents’ divorce is influenced by the quality of parental care [14]. Studies looking at the parent–child relationship as an important factor for the functioning of siblings after divorce use the theoretical frameworks of attachment theory, social interactions theory and parental differential treatment [21,22,23,24]. Children securely attached to their parents tend to have a positive relationship with their siblings [25,26,27]. Research by Patterson [28], also confirmed by other reports [29,30,31], referring to the processes of social learning, shows that families with conflicts in mutual interactions create an environment in which children also shape conflicted relationships with each other. Noller [32] describes this phenomenon as “the transmission of interaction patterns,” emphasizing that children learn to behave towards each other based on how they interact with their parents. It is also consistent with the concept of “Internal Working Models”, developed in the framework of attachment theory [33]. Finally, parental differential treatment (i.e., unequal meeting of children’s needs) was found in many studies to be a factor explaining sibling conflicts [32,34,35,36]. In addition, it is emphasized that in situations of a family crisis (e.g., economic or related to divorce) and increased stress experienced by the parents, parental differential treatment may become more intense [37]. In order to explain the impact of such situations on the functioning of siblings, it is necessary to define how children perceive parental care as fair or unfair because it turns out that conflicts arise in the sibling subsystem only when parental differential treatment is perceived as favoritism [38]. This means that children can distinguish between legitimate parental differential treatment and obvious favoritism. Finally, it is worth noting that the link between parent–child relationship and sibling relationship is two-way, and what happens between the children can influence their individual relationship with the mother and father and the parental relationship [21,39].

Sibling relationships, rooted in shared childhood experiences, are most likely to be the longest-lasting of all family relationships, and, when positive, they become an important source of support throughout life [40,41,42], also in a family crisis [43]. The conclusions from the literature review implicate that there is no unequivocal assessment of the impact of divorce on sibling relationships. Research on marital conflict and parental divorce as sources of stress for family members indicates their negative impact on sibling relationships [44,45]. The breakdown of the family increases the competitive behavior between children [46] and fosters the intensification of children’s conflicts of loyalty towards their parents or siblings [47,48]. According to the congruence hypothesis, the relationship between the children mirrors the relationship between the parents [25,49], which can be explained by the modeling process or the influence of stressors such as parental conflict on the deterioration of relations in the sibling subsystem.

The relationship between sibling hostility and rivalry with parental conflict is mediated by hostile parenthood [50]. On the other hand, Bank and Kahn [51], Jenkins [52] and Lanthier and Furman [53] claim that having siblings can serve as a protective factor in the face of stress affecting the family. The compensation hypothesis [51] suggests that a strong sibling bond is an important source of support for children in crisis situations, such as parental conflict or family breakdown. The consistency of the sibling subsystem and the closeness between brothers and sisters increases after divorce [54]. Moreover, children post-divorce prefer closer relationships with each other compared to children in intact families [55], as they feel that a brother or sister is the only other person who can understand the feelings linked to the family breakdown, and the siblings are one of the few elements of the system that can remain unchanged [43]. Siblings are the source of the “ontological sense of security” and the sense of continuity of the self during a change in the family environment, meaning that they can act as “transition objects” when parents exchange care and children change their place of stay [56]. Moreover, closeness among siblings better explains the positive adaptation of children to divorce than in the case of conflict and negative consequences [57].

In addition to reports suggesting a positive or negative impact of divorce on the strengthening of bonds between brothers and sisters, there also are reports that indicate strong feelings and simultaneous attitudes of hostility and closeness of siblings towards each other [58,59], which further complicates the predictions concerning the change dynamics in the sibling subsystem in the face of divorce. Typology of relations among siblings provided by Sheehan et al. [58], based on work by McGuire and colleagues [60], shows that among adolescents from divorced families, the affect-intense group is overrepresented, meaning that their relationships include intense closeness as well as conflict. Adolescents participating in the study reported that the intensity of both dimensions was linked to the older siblings’ caretaking of the younger ones, which created closeness, but at the same time, was a frequent source of conflicts (older siblings corrected behaviors of the younger ones, who were then dissatisfied with their brother or sister interfering with the parents’ duties). Separate from other family relationships, siblings’ inner world functions on many levels and takes many forms. The three dimensions of the sibling subsystem (common rituals, play and conflict) [61] are often disrupted in the case of divorce. Rituals help cope with stress and, according to family therapists, are important for well-being and vice versa—their lack makes it difficult to adapt to life changes. This aspect is rarely discussed in the research, and as reported by Isaacs [62], in the first year after the parents’ separation, children’s bedtime rituals were often disrupted, which was related to behavioral problems.

During a divorce, sibling ties may continue and strengthen by maintaining rituals unique to this relationship [61]. Similarly, play serves an important role in children’s functioning and may be therapeutic. As such, it should raise concern if the parents inform that this sibling activity disappears around the time of divorce (from the authors’ clinical practice). As the review of research shows, the frequency and intensity of conflicts between children increases, but they may be of a different nature and purpose. Sibling conflict is a form of communication and play; it serves to regulate the distance or seek revenge. Often unequivocally assessed as negative by adults (parents, experts), it can play an important role for siblings who stay in touch with each other this way and regulate their emotions during arguments or fights.

Research by Jenkins and Smith [63] shows that in a family crisis, the mere presence of a brother or sister is not enough, but positive relations among siblings are a protective factor. However, close relationships in the children’s subsystem may be a source of conflict when the older sibling reminds the younger one of the need to remain loyal to the biological parent who is not the primary guardian. This happens during family reconstitution when the younger child shows positive feelings towards and becomes closer to the new partner of the parent who is directly caring for them, while older children need more time to adapt to changes in the family [64]. It turns out that when siblings stay together after their parents’ divorce, children often assume pseudo-parental roles [65,66]. One of the strategies for dealing with separation from the parent and the related gap in the family system is parentification. Parentification is defined as a pattern of family interactions in which a child or an adolescent assumes roles and takes on the responsibility assigned to adults in their culture, while the position of the parent in such a family is weakened [67,68]. In children and adolescents, performing age-inadequate tasks may cause both internalizing behaviors, such as depression and psychosomatic symptoms, and externalizing behaviors, such as behavioral and personality disorders [69,70,71]. However, in some circumstances, parentification may engender feelings of self-efficacy, competence and quality relationships between siblings may reduce the negative outcomes of parentification [72].

In a small number of studies on sibling relations in a divorce-related situation, there is a scarcity of research on children who were separated as a result of their parents’ decision to split up. Observation of the functioning of such families, resulting from the work of the authors of this publication as psychologists providing expert opinions for the courts, suggests that this is a highly unfavorable situation for children. It is so especially when, due to the lack of parental agreement, the children often change their place of residence. This way, they are deprived of the source of support from their brothers and/or sisters, face family breakdown and separation from one of their parents, and they also lose daily contact with their siblings. They cannot worry together, and they feel lonely [47,73]. When staying together during and after divorce, it is important for children that their siblings are there as they have gone through similar events and may have similar experiences [74]. Children living apart lose the opportunity to share the burden of trying to remain loyal to each parent [75].

One way is for each sibling to remain loyal to a different parent so that both adults have an ally in the family [76], which may, unfortunately, increase the emotional distance between children [77]. This relationship pattern is referred to as “split-parent identification” [78]. At the same time, the question arises whether sibling separation, in such a way that some children remain under the care of one parent after divorce, and some under the care of the other, does not constitute a factor protecting against parentification (i.e., performing adult tasks by children in order to minimize the consequences of the lack of a parent).

The aim of the conducted analysis was to assess the causes and consequences of sibling separation due to parental divorce (the first case concerns the period around the time of divorce, and the second case is the period after divorce and the ongoing crisis related to lack of parental agreement in the context of the two reconstituted families). For both families, the lack of parental agreement is a risk factor, and what follows is the lack of arrangements and no permanent decisions regarding the place of residence of the children.

## 3. Materials and Methods

In the course of the diagnosis conducted by the authors as part of court procedures, the psychological functioning of children was assessed with the use of inter alia (in addition to the standard methods such as interview, observation and questionnaires recommended by the Polish Psychological Association and analysis of data contained in court case files) diagnostic play as a method that is emotionally non-burdensome and interesting to children.

Traditionally, play therapy has been used in individual work with children with various types of difficulties experiencing crisis situations in their lives [79,80,81,82]. The diagnosis of family functioning through play with a child presented in the case report is a combination of techniques traditionally used in therapy through play and the assumptions of systemic family therapy and the methods used in it [83,84,85,86]. The analysis of games was based on the structural theory of Salvadore Minuchin [87] and carried out on the basis of the criterion of cohesion, hierarchy and boundaries in the family system. The dimension of cohesion is related to the ties connecting family members, hierarchy relates to the clarity of dependence and order in the family system, and the boundaries are expressed through flexibility/rigidity and openness/closure of the family system to exchange with the environment. The assessment of the content revealed in the play was analyzed in the context of the individual situation of the child by experienced clinical psychologists. The record of the play was standardized through a protocol in which the diagnostician records the child’s behavior and the choices made by him.

The use of this method of psychological diagnosis was particularly important in the described cases due to the generally elevated level of tension and stress among the children related to a difficult family situation. Play is a valuable tool in the diagnosis of emotional ties in the family and in children’s attitudes towards family members because the “play” situation is natural to children whose life activity is largely related to playing. The content and form of play can serve as a diagnostic tool because such a situation is not threatening to the child, which allows the disclosure of real attitudes and relationships. The purpose of using playing is to bring out their thoughts, experiences and wishes in an indirect and less intrusive way than during a conversation. In the described cases, we used selected methods of diagnosis through play derived from the diagnosis of family ties of children and adolescents—a diagnostic tool published and used throughout Poland [88]. It is a collection of 15 diagnostic games developed by the authors, based on over 20 years of experience as psychological experts diagnosing families for the justice system. A short description of the diagnostic games used in the described cases can be found in Appendix A. The selection of games used in the diagnosis process should be adapted to the age of the child and should also serve a specific purpose, which, in the case of the presented research, was determined by the court. The aim of the study and thus the decision to choose the games from the set depends, therefore, on the questions posed by the court and on the type of case: divorce, regulation of contacts between children and parents, childcare.

## 4. Results—Case Studies

### 4.1. Case Study 1—Around the Time of Divorce

Family members: mother, 28 years old; father, 31 years old; son, 9 years old; daughter, 6 years old; daughter, 2.5 years old.

Family: The first years of marriage, concluded 10 years ago, are perceived by both spouses as satisfactory. The crisis in their relationship occurred about 4 years before the beginning of the formal divorce proceedings. According to the woman, the crisis was related to the husband rediscovering his family of origin (after several years of no contact), and according to the husband, it was linked to her acting aggressively (verbally and behaviorally), also toward the children and especially towards the middle child—the 6-year-old daughter. The spouses did not try to salvage the relationship, did not look for a solution to their crisis, and separated 1.5 years before the diagnosis took place. The man and the 6-year-old daughter left their place of residence and moved to his parents’ place. Currently, the son and younger daughter are under the direct care of the mother, and the older daughter is under the care of the father. For six months, he has been in a relationship with a woman who has two children from a previous informal relationship—a 5-year-old daughter and a 4-year-old son.

The purpose of the expert opinion ordered by the court was to answer the following questions: What are the emotional ties between parents and their minor children? What is the parenting capacity of each parent? Is the legal dissolution of the marriage not against the best interest of the children? Are the children prepared for their parents’ divorce? In the future, how will the contacts of the minors with the parent they will not be living with be set up?

#### 4.1.1. Games Used during the Diagnosis of the Couple’s 9-Year-Old Son

The aim of the first game (QUEUE TO THE HEART) used for the diagnosis of the boy was to see his emotional ties with people who were important to him and the hierarchy of those people. During the first game, when asked who was important to him, the boy mentioned family members in the following order: maternal grandmother, maternal grandfather, mother’s brother (uncle), mother’s brother’s wife (aunt), mother’s sister (aunt), mother, father, sister (6 years old) and younger sister. The hierarchy of important people was as follows: maternal grandfather, maternal grandmother, uncle and aunt (mother’s brother and his wife), older sister, younger sister, mother and father. In both phases of play, the boy first mentioned the members of his mother’s family of origin with whom he lived during his early childhood and where he lives now, after the separation, as the mother moved with her two children to her parents. Throughout the boy’s life, his grandparents were involved in helping his parents with his upbringing.

His choices were a sign of a positive bond with them; however, placing parents and siblings further may have been a sign of emotional ambivalence linked to the crisis in the family and the tension in the parental relationship. Such a positioning of the members of the primary family may also be a signal of a conflict of loyalty that he felt due to the separation of his parents, their conflict and the attempt to create a coalition with children against the other parent by both the father and the mother. Both hypotheses can be supported by the assumption that in the current family crisis, the parents were not a source of support and a guarantee of a sense of security. In the second phase of play, younger sisters appeared before the parents in the hierarchy of people participating in his emotional life. This shows a strong bond with siblings but also a sense of greater freedom in expressing positive feelings towards them than towards parents who are in conflict.

The purpose of the third game (WHO CAN HELP?) was to assess the hierarchy in the family in relation to the psychological resources needed to cope with problems. In the first phase of the game, when asked about people who would help him solve his problems and who would provide him with support, he mentioned his mother, maternal grandfather, maternal grandmother and older sister. Despite the ambivalence of feelings or the lack of a sense of freedom in expressing love toward the mother, the boy perceived his immediate guardian as a competent problem solver. This may be a signal of the proper fulfillment of the child’s needs by the mother, who has been raising two children on her own since the separation. Next, there were the maternal grandparents, whose house he lives in and who provided him with emotional support as well as support with everyday duties. The boy did not mention his father, with whom he has limited contact, among the people helping him with problematic situations. However, his younger 6-year-old sister appeared as a potentially helpful person, which can be understood as him missing his sister, also in her role as a source of support in a crisis. The siblings remained close to each other during their parents’ marital quarrels, and their presence could give them a sense of security. In the second phase of the game, the boy was asked about people who would be approached by his 6-year-old sister. He listed their mother, maternal grandfather, maternal grandmother and father. Such a choice confirms the perception of the mother as a competent parent, helpful in solving problems, and the maternal grandparents as a source of support. The choice of the same people as in his case could imply a projection of his situation onto his sister’s and his difficulty in adopting her perspective. However, the choice of the last person—the father—as helpful for the sister in problematic situations, in the absence of this parent in his own choices, showed that he was aware of his and his sister’s different life situations linked to living separately, under the care of different adults.

#### 4.1.2. Games Used during the Diagnosis of the Couple’s 6-Year-Old Daughter

The first of the games (“MONSTER” RIVER”) was used to assess the strength of her bond with family members and her perception of their ability to cope with difficult situations (as perceived by the child). In the initial phase of the play, the girl mentioned the following family members: father, brother (9-year-old), mother, younger sister, paternal grandmother and paternal grandfather. The game showed that the girl, despite the separation of parents, perceived the family as coherent, with clear boundaries—since she first mentioned the people who belong to the primary family. However, the order in which she listed the immediate family members may indicate that she perceived the marriage subsystem as broken, and in the face of the parents’ conflict, she was closer with her father, whose care she had been under for two years. It also seems that the girl communicated the preferences for one of her parents more openly than her brother—she expressed positive feelings towards her father and distanced herself from her mother. This may be related to impulsive behaviors directed by the mother towards the girl. The mother did not deny those behaviors, stressing, however, that they were never intentional and occurred during quarrels with her husband when the children tried to interfere in the parents’ conflict. However, distancing from the mother may not be the expression of the girl’s own feelings but rather the result of the actions of the adults under whose care she remained.

During questioning of the girl in the court, in the course of the case, a phrase similar to the one in this study (in the next game WHEELBARROW FOR PROBLEMS) appeared, which was suspected of having been prepared beforehand and learned, and not resulting from the minor’s direct experiences. In both phases of play, she placed the brother high in the emotional hierarchy, as she missed him, and his absence was particularly painful to her; a high place was also allocated for the sister. This was probably due to the strong bond between the siblings, who supported each other during their parents’ conflicted marital relationship. The girl admitted in an interview after playing that she moved her brother and sister first because they are children; as such, they are the weakest and therefore in need of quick help. It was an expression of her caring behavior but also a metaphorical representation of the situation of the children in the observed family.

The aim of the second game (WHEELBARROW FOR PROBLEMS) was to diagnose the types of problems that the child experiences as well as the importance of these problems and their order of importance. When asked: “What is a problem for you, what are you worried about, what bothers you?” spontaneously and quite quickly, she mentioned her troubles and matters that she worried about: *Parents quarrel and fight*, *we do not live together*, *I do not live with my brother* (9-year-old), *Daddy has little money*, *Mommy hit me in the tummies*. When asked to think about what worried her the most, she arranged the problems and then symbolically removed them in the following order: *I do not live with my brother* (9-year-old), *Daddy has little money*, *Parents quarrel and fight*, *we do not live together*, *Mommy hit me in the tummies*. The game showed that the separation of her parents and their conflict, and most of all, separation from her brother, were difficult for her to process, despite the fact that she felt that her needs were met when living under the care of her father and that she partially adapted to living in the house of her paternal grandparents. This indicated a lack of acceptance of the family breakdown, a lack of preparation for the parents’ divorce due to their strong conflict and a lack of discussion about the children’s situation after the separation in a way that met their needs. The appearance of the problem “Daddy has little money” can indicate her inclusion in adult matters and burdening herself with her father’s troubles. She also said, “Mommy hit me in the tummies”. This phrase, described in the above game, may undoubtedly indicate the mother’s aggressive behavior, but the particular phrasing of the sentence and its repetition in the course of the case raises suspicions about its spontaneity.

#### 4.1.3. Conclusions from the Diagnosis (Based on the Games)

The emotional bonds of parents and minors are preserved, but due to a strong marital conflict, they are not formed properly. The boy is emotionally distancing himself from both parents, which is an irregularity and a way to resolve the loyalty conflict. On the other hand, observation of the girl’s play pattern reveals that she resolves the loyalty conflict by choosing the father and getting emotionally close to him and his family of origin while distancing herself from the mother by emphasizing her negative behaviors (which the girl could have experienced directly as the object her mother used to release tension, or witnessed parents aggression towards each other during their arguments).

In the scope of caring individually for the children who live with them, both parents are able to meet the vital needs of the children. On the other hand, the parenting capacity of both the mother and the father is reduced due to their intense conflict. Parents’ mistakes in the past include exposing their children to seeing their aggression toward each other, as well as their negative behaviors towards children who tried to interfere in the conflict. At present, however, the lack of desire to communicate and the separation of siblings as the only solution to the conflict prevents adults from preparing their children for a possible divorce. The tension in the spouses’ relationship also makes it difficult for them to cooperate, which they should aim for in order to minimize the emotional consequences of family breakdown, primarily for the children. Deficits in the parenting capacity of the mother and the father warrant a limitation of their parental authority. Children acutely experience a lack of contact with each other. Therefore, above all, contact between the children should be made possible in a way that allows them to meet in a relatively emotionally favorable atmosphere.

### 4.2. Case Study 2—Contact Order

Family members: mother, 38 years old; father, 39 years old; son, 16 years old; daughter, 10 years old

Family: The children’s parents split up 7 years ago, after 9 years of marriage. By the court’s decision, after the divorce, the children remained under the direct care of the mother. Both partners started new relationships—the mother has been in an informal relationship for 5 years; the father got married 4 years ago, and he has three children with his new wife (all daughters 5, 4 years old and the youngest, 1 year old). Two years ago, at the request of the son, but also with the consent of the parents, the care of the boy was taken over by his father. The father applied to the court for the validation of this situation and his care for the son.

The purpose of the expert opinion ordered by the court was to answer the following questions: What is the state of the emotional ties between parents and their children? What is the son’s relationship with his father’s wife, half-siblings and mother’s partner? What is the state of the relationships between siblings? What is the level of parenting capacity of both parents? What is the son’s position with regards to living with the mother or father, and is it in his best interest to take his will into account?

#### 4.2.1. Games Used during the Diagnosis of the 16-Year-Old Son

**Figure 1:**
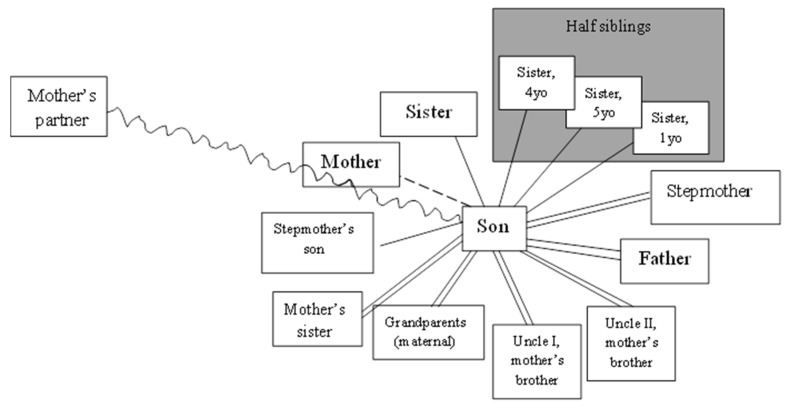
Quality of son’s relationship.

During the first game (PICTURE OF THE RELATIONSHIPS), the boy’s task was to create a map of family relationships—emotional ties between family members and conflicts (as perceived by the child). During the conversation with the boy, a drawing was created (Figure 1) in which he placed the members of his original family (father, mother, younger sister), people from the reconstituted family in which he is currently being brought up, as well as members of his mother’s family and her partner.

The drawing illustrates the quality of his relationship with those involved in his life. In his opinion, he has a very good relationship with his father, stepmother and people from his mother’s family of origin. They live in the same town as the mother, and the boy meets them when he is visiting his mother. He especially respects his grandparents because he believes that it was them, and not his mother, that raised him during his early childhood. He appreciates his mother’s brothers for introducing him to various activities, thanks to which he could learn the profession of a construction worker, and during the summer holidays, he was financially rewarded for his help and work under their supervision.

He also has a strong relationship with his mother’s sister, whom he sees as a supportive and trustworthy person. He also has a good relationship with other children in the family—his younger sister, stepmother’s son and step-sisters from his father’s current marriage. During the diagnosis, the boy emphasized that he did not favor any of the siblings. At the same time, he is aware of the fact that he does not take care of the relationship with his younger biological sister, who misses him and especially misses living with him. The fact that he does not stay in close contact with siblings may result from the development phase and the increased importance of contact with peers. He has a distant relationship with his mother, while he is in conflict with her current partner. The boy resents his mother’s partner, who, according to him, wrongly accused him of hitting his mother. According to the boy, during visits to his mother’s, he avoids her partner and does not seek close contact with the mother either. However, he regularly visits her at her place of residence, which he explains with his desire to stay in touch with people from his mother’s family of origin. It can be assumed that the boy’s distance and ambivalent feelings towards his mother may be rooted in the conflict with her partner. Maintaining a strong bond with the mother’s family of origin may be, in his current situation, a way for him to maintain contact with his mother as well. It is also an expression of the willingness to remain close to her, which can be more difficult to express and show directly due to the developmental phase and loyalty to his father, who is his immediate guardian.

The goal of the second game (TWO HOUSES) was to diagnose the parental competence of parents in the child’s perception and the bond between the child and the parents. During the diagnosis, the boy allocated the sentences describing his parents’ guardianship and educational competence to each of the houses in which he lives:

Father’s house: In this house, they take care of me; It is fun in this house; There are quarrels in this house; In this house, they help me when I have a problem; Children are treated fairly in this house; In this house, adults behave consistently with children; They express tenderness in this house; In this house, I feel important and appreciated; In this house, compromises are sought to solve problems.

Mother’s house: In this house, they take care of me; There are quarrels in this house; They express tenderness in this house; In this house, I feel important and appreciated.

The choices made by the boy revealed that he perceives both the father’s and the mother’s house as places where he is taken care of, and he does not feel neglected or underestimated. He observes that in both houses, relatives show positive feelings for each other, but also during visits to his mother’s house, as well as at his father’s house, he witnesses conflicts between family members. Revealing both positive and negative aspects of the emotional atmosphere in both houses indicates a willingness to remain objective in the assessment and a lack of tendency to idealize either caregiver. By analyzing the assignment of sentences defining adults’ competence in supporting him and helping him solve problems, it can be concluded that in this respect, he places a higher value on the father and his current wife higher than on the mother. During the interview, he stated that his stepmother was the person who helped him with his studies and discussed with him the choice of high school. He also feels that the children are treated fairly in the father’s house, and despite the fact that he is the father’s child from a previous marriage, the stepmother’s child from her first relationship and three children in common are brought up in one family, neither of them is favored. He stated that he wants the father to take over as the main caregiver because, for example, the mother and her partner treat his younger biological sister better than him.

#### 4.2.2. Games Used during the Diagnosis of the 10-Year-Old Daughter

**Figure 2:**
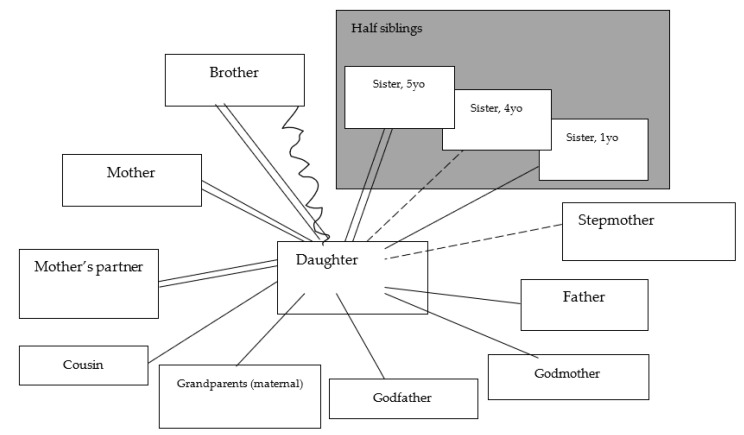
Quality of daughter’s relationship.

The girl, like her older brother, made a map of family relationships—emotional ties between family members and conflicts (in the child’s perception) Figure 2.

The picture of family relations reveals that for her, just like for the older brother, family members include parents, biological brother, stepmother, mother’s partner, step-sisters, as well as people from the mother’s family of origin: grandparents, godparents and a cousin (son of the mother’s sister). She feels the strongest connection with the mother and mother’s partner, whom she assesses very positively as a guardian. However, she fears that her positive feelings toward her mother’s partner will be met with a negative reaction from her father, and therefore in his presence, she tries to control herself and behave in such a way so as not to reveal her close relationship with her mother’s partner. The emotional bond between the girl and her father is also preserved. She misses her father and would like to spend more time with him. It is important for her that he spends time only with her during her stay at his house. She does not perceive her half-sisters as rivals, although she differentiates the quality of the relationship with them—she feels most positive about the eldest one, remains in good relations with the youngest, and sees the relationship with the middle half-sister as distanced. The girl has a strong relationship with her biological brother. However, in her perception, this bond has traits of a conflict because of the brother’s attitude towards her and his reluctance to contact her more often and become closer. The girl misses her brother even more than she misses her father. In the face of crises in the family (parents’ conflict, their separation and divorce), for her, the bond with her brother was a stable element of the family system; therefore, the transition of her brother to their father’s care could be a particularly painful change for her. This change meant that she lost everyday contact with her siblings and found herself in the position of the only child in the family. Partly, she may feel privileged because of this, hence her need for her father’s exclusive attention when she spends time with him. However, on a daily basis, she has lost the closeness and source of support that the presence of a brother or sister provides to children whose parents divorced. The girl has a good relationship with people in her mother’s family of origin, which is an additional source of support for her in the face of past family crises. Close contact with a cousin can compensate for the loss of closeness with a brother. The stepmother’s son does not appear among family members, and she marked her relationship with the father’s wife as neutral, which may mean ambivalent feelings towards the person with whom the father became involved and perceiving her in the past as the reason for the separation of her parents and the person whose presence prevented her mother and father from reconciling.

In the second game (MEDALS), she assessed the parental competence of the mother, father and mother’s partner and the degree of meeting the needs of each parent.

She positively perceives the mother as the direct guardian, as well as her partner, in some areas placing him even higher than the biological parents (e.g., help with homework, support in solving problems). With the area “Help with homework”, the girl decided not to give her father a grade because, as she stated, she does not do her homework during visits to her father. She assessed the father as slightly lower than the mother and mother’s partner in terms of parental competence, thanks to which the child feels that they are important to the parent. A lower grade for the father in terms of fair treatment of children, showing his daughter love, and organizing free time may be related to the girl’s feeling, described above (when playing PICTURE OF THE RELATIONSHIPS), that after her parents split up and the father started a new family, she lost his attention. However, she feels that adults have positive feelings towards her, which they openly express.

#### 4.2.3. Conclusions from the Diagnosis (Based on the Games)

The bond of children with both parents is preserved. The boy’s emotional bond with his father is strong and develops correctly. The boy’s feelings towards his mother are ambivalent, probably also due to his conflict with his mother’s partner. The girl has a strong relationship with her mother. She has a good relationship with her father, although she experiences a deficit of his attention when she visits him in his place of residence.

Both children include their parents’ current partners among the people they are close to, which shows that they have adapted to a certain degree to the situation after the divorce and to the reconstituted relationships of their mother and father. On the one hand, they do not consider their parent’s new partners as people with whom they remain in very good relationships—they keep their distance (stepmother—girl) or remain in a quiet conflict (mother’s partner—boy). On the other hand, they became emotionally attached to the parents’ partners with whom they live—the girl to her mother’s partner and the boy to his father’s wife. The relationship of the biological siblings is complex and perceived differently by the girl who misses her brother and the boy who, in his opinion, has a good relationship with her sister but minimizes the importance of contact with her in favor of peer relationships. The difference in the assessment and the need to maintain the brother–sister bond can be explained by the age of the children and different needs resulting from the development phases, but also by their current situation—she has become the only child in the family where she is growing up, while he lives in a home where four children younger than him are brought up.

Adults who participate in taking care of them are generally assessed positively by both children. However, they emphasize the higher competence of the parents and parents’ life partners who care for them directly. In their assessments, they also express the deficits they feel in contact with adults as guardians—she signals that her father is not paying her enough attention, while the boy may expect greater support and help in solving problems from the mother.

The boy adapted to life in a family reconstituted by his father, in which he has been growing up for two years. He feels that his needs are properly met there. The results of the diagnosis are in support of the child’s decision, but the parents should clarify the rules for their parental cooperation, and the father, who is an authority figure for the boy, should help his son work out the relationship with the mother. The next step would then be to work on the boy’s relationship with his mother’s partner so that the contact with the mother and the time spent in her home would be fully satisfying for him.

## 5. Discussion

We presented the results of the process of diagnosing families during divorce, in which play was the key tool for examining children. The first goal was to analyze the reasons for the separation of siblings whose parents were in conflict during the separation (first case study) and after the separation (second case study), as well as to assess the functioning of the children resulting from the family breakdown, and the decision to separate them from siblings. The analysis allowed identifying the areas of sibling functioning, which should become the subject of diagnosis when working on expert opinions in divorce cases, or cases establishing contact between parents and children. The second aim of the report was to assess the effectiveness of using play as a diagnostic method in a situation that is a source of stress for the child (family breakdown) and which causes tension (the diagnostic process in which this topic is discussed).

Separation of siblings during their parents’ divorce/family breakdown is one of the most serious legal and clinical diagnostic problems and always requires consideration of all consequences, both immediate and distant, for the children who are affected by this situation. This type of shared custody happens when one parent has legal and physical custody of one or more children and the other of another child or children. This situation is much more common in families with older children.

We deal with three perspectives when resolving such cases: legal (i.e., what is possible under the applicable family law), parental (expectations and sometimes earlier agreements between parents) and children’s (which may significantly differ from the others; moreover, children from one family may have different perceptions of the same situation).

Judges are generally reluctant to separate siblings, arguing that children derive numerous instrumental and emotional benefits from growing up with brothers and sisters [89]. As pointed out by Nichols [90], the most important problem with regard to the division of care is that the separation of siblings after divorce significantly reduces the time spent together, negatively affects the level of closeness and quality of ties and limits the possibilities of free, direct communication. This can lead to a reduction in the overall quality of the relationship and the weakening of their bond, the significance of which is an important reason why separation of siblings following divorce is a solution rarely chosen by the courts. The courts recognize that constant access to close family members, including siblings, is a right that is in the children’s best interests.

The decision to separate the children is most often, but not always, a consequence of the parent’s choice to split up and share the custody of the children, made prior to the divorce. There are many reasons for such a solution, and the most common ones include clear differences in the relationship between parents and each child (the level of closeness, freedom and quality of communication, common interests, etc.), a way of solving conflicts among siblings and treating such a solution as fair from the parents’ perspective. As in the case of alternating custody, the question arises whether this solution is beneficial for children not only in the short term but also in the long term. The analysis of these consequences is an important task of court experts in family matters, and therefore when conducting a clinical diagnosis commissioned by the court, court experts take into account both the short and long-term consequences of the separation, the reasons for which it is requested, and other ways of solving the problem (for example, of severe sibling conflicts), as well as ties between individual family members, and parental competences.

Smyth et al. [91] describe three groups of conditions, the inclusion of which seems justified in court cases for separation of siblings: objective (economic and living, the distance between parents’ houses, organization of work, entertainment and time devoted to the child); individual and developmental related to the child (their temperament, health, talents and interests, special needs and adaptation skills); parental and concerning parental relationship and the wider family environment, especially the parental competences, their adaptability, mutual trust and cooperation, the flow of information between them, the coherence of educational environments, ties with siblings—including siblings from new relationships, support from the wider family, including new partners. In cases regarding the separation of siblings, the analysis of the dynamics and direction of changes in the relations of siblings in the situation of divorce can be added to the factors identified by the authors. The literature review shows two opposing positions regarding the consequences of parents’ divorce on the quality of relations in the children subsystem. The first of them, supported by a smaller number of studies [65,92], shows that siblings become closer when faced with a difficult situation. Numerous scientific reports support the second perspective, showing that children do not support each other, become distant and more hostile to each other, and the conflict between them increases [17,68,93,94,95]. Separation of siblings used as a solution in the situation of parents’ divorce seems to be another risk factor for the growing distance between the children who will not have contact on a daily basis, and the regularity of their contact may—to a large extent and especially so in the case of younger children—depend on adults. On the other hand, the review of the modest literature on separation of siblings in divorce cases and the case study presented in this report also helps identify some elements of such a solution that may protect children from excessive mental strain. The risk factors undoubtedly include (1) weakening of ties among siblings as a result of lack of daily contact and living together; (2) change in the constellation of the sibling subsystem in terms of the number of children and their position according to seniority, and the impact of this change on the quality of mutual relations; (3) the absence of everyday rituals and playtime.

Ad (1) Sibling separation is generally perceived negatively by children, mainly due to the fact that it is another trauma (loss) that they experience as a result of the family crisis. Although children should not be used as substitutes for adults, our observations confirm the reports [58,61] that siblings may provide each other with a sense of security and continuation when their parents divorce. In the first of the presented cases, in a game showing the hierarchy of people who are important in their lives, the children chose siblings before the other parent. This could mean not only that they missed their sibling but also the loss of the possibility to express feelings related to the parents’ divorce, which is often easier and more spontaneous towards the siblings than adults. It is also worth remembering the particularity of sibling relationships related to the fact that the mutual hostility and conflict do not negate the fact that they can be an important source of support for each other. Frequently, children discover a safe space to express all and various emotions related to the separation of their parents in the sibling subsystem. The analysis of the presented cases confirmed that in the situation of the divorce and especially in the face of separation, children could remain in affect-intense relationships with each other [60]. Those are characterized by a high level of both positive ties and hostility. However, the lack of conflict is not a sign of close ties in the sibling subsystem, but it may signal a gradually deepening emotional distance resulting from the physical separation of children. As shown by the authors’ clinical experience, the weakening of the bond with siblings is a risk factor for the development of irregularities in psychological functioning due to the loss of support and oftentimes the authority of the older brother or sister in the case of the younger child. Therefore, when establishing contact between children and their parents after divorce, solutions that serve to maintain and strengthen sibling ties should be considered. In addition, while in the case of a parent with whom the child does not live, their contact is usually regulated (by parental agreement or court order), in the case of siblings, this contact is again conditioned by whether the adults who divorced, cooperate in the matters related to the children.

Ad (2) The separation from siblings after divorce, when a relationship reconstruction by parents occurs, means a change in the child’s position in the family, with age and gender having additional significance in these changes. In the case of a divorce, often one of the children—the older one, often a teenager, distances themselves from the family and other children, wants to organize their feelings by themselves, and withdraws from family relations. Often the older child understands better the real reasons for the separation of parents and the sources of their conflict and wants to protect the younger ones from this knowledge. Sometimes the family, including younger siblings, is even rejected, and the younger ones feel rejected and lonely [45,65], as in the second of the discussed cases. The brother did not perceive the relationship with his sister as a conflict, while the sister saw her brother’s distance from her as an expression of resentment and anger. Visibly the children had different perceptions of their relationship. Further, due to the persistent disagreement between the parents, the age difference and the brother and sister being in different developmental stages, all of which may deepen the distance, children’s ties are at risk of weakening.

Parents perceived the situation of their children in a different way—during the study, they explained their decision to maintain the situation of separating the children by the fact that they were not emotionally close. As indicated in the literature [45,65,96], in the system consisting of an older brother and a younger sister, distance and conflicts appear more often and may increase over time; therefore, such a constellation of siblings may require special attention during the diagnostic process. Another experience, which might be difficult for some, is suddenly occupying the position of the only child as a result of the siblings’ separation, which happened in the second described case and increased the feeling of loneliness of the examined girl. The literature, as well as our own analyses, confirm that children’s gender remains an important factor related to the consequences of divorce when siblings are separated. Sisters—more often than brothers—provide comfort to their siblings, especially their other sisters [97,98]. In the first of the presented cases, the eldest sister expressed her regret that she did not live with her brother and, in the form of play, indicated that her younger siblings were those people in the family who required special care.

Ad (3) When separation occurs, children lose the possibility of everyday contact with each other and of the feeling that there is someone else who is experiencing a situation similar to their own. As visible in the situation of children presented in the report and the literature review, the presence of siblings is a source of a sense of security, even when they do not talk about a difficult family situation. Research by Bush and Ehrenberg [65], who interviewed young adults whose parents’ divorced in childhood, showed that most of them felt that the presence of a brother or sister helped them cope with the situation; even if it took very subtle forms such as sitting side by side and watching your favorite TV program together. The longing expressed for siblings, especially by the sisters in both discussed cases, is probably longing for joint play, family rituals and even the possibility of arguing. Eno [61] drew attention to the disturbance of these dimensions in the sibling subsystem due to the divorce, and in the cases of siblings separation, there is a risk that they disappear completely.

Among the protective factors—the elements of the situation that can reduce the emotional burden of the conflict and parents’ divorce, the following can be considered: (1) separation of older siblings from younger ones, which may protect against parentification; (2) reduction in tension in the sibling subsystem as a consequence of lowering the level of conflict of loyalty towards parents; (3) weakening of the feeling of unfair treatment and favoring of siblings by the parent or their new partner.

Ad (1) The literature, the case studies and the clinical experience of the authors show that in families in a situation of divorce, older siblings often assume the role of caregivers towards the younger ones, for example, by trying to protect them from parents’ arguments or by interfering in the fulfillment of chores by younger children, to avoid yet another excuse for conflict. The younger siblings did not like the involvement of the older ones in their lives while appreciating the efforts of a brother or sister who cared for them and tried to maintain a good atmosphere in the family. The above observations are consistent with the research by Noller and her colleagues [59]—in the face of the parents’ conflict from before the separation, during the divorce and after its formalization, the younger children did not value the caring behaviors of their older siblings towards them and described them as “overprotective”. On the other hand, siblings’ separation may be the cause of loss of the “guardian”, as was in the second described case. The younger sister openly expressed she missed her older brother, who lived with their father after the divorce, while she stayed with their mother. The boy did not respond to the signals sent by his younger sister, expressing her need to spend time together. From his perspective, the adopted solution could be a way of refusing the pseudo-parental role. Moving to his father’s house freed him from the risk of parentification. In the first case, the oldest sister also displayed beliefs and play behaviors that indicated her sense of responsibility for younger siblings who should be protected.

Ad (2) Separation of siblings used as a solution by conflicted parents usually results in a reduction in tension (unfortunately often short-lived) among all members of the family system, including the sibling subsystem. In the diagnostic process, when analyzing children’s conflict before, during and after the possible separation of parents, it is important to distinguish children’s aggressive behaviors that are a way of relieving tension from a conflict that reflects the relationship of adults and is an expression of loyalty of the child towards the chosen parent. The first situation seems to be typical for siblings, as long as it does not pose a threat to their safety and health, also in the situation of divorce. In a study by Bush and Ehrenberg [65], young adults, when retrospectively assessing their relations with siblings, admitted that the tension they experienced, which was linked to the separation of their parents, caused them to be aggressive towards their younger siblings. The younger brother or sister was not perceived as guilty of the situation, but they were available and were an easy outlet for negative emotions. It is, therefore, once again, an affect-intense relationship rather than hostility towards a brother or sister. The second situation is more threatening for the sibling relationship and their functioning, and the persistent parental conflict can contribute to the increase in distance and hostile attitudes of children towards each other. As our observations show, in a situation of a persistent or increasing conflict between adults, children often resolve their loyalty conflict by choosing one of the parents and, by becoming their ally, place themselves in opposition to the other parent and siblings who remain under their care. The cases described in the report showed that after separation, the children clearly identified with the parent who was their direct guardian, but at the same time maintained a bond with the other parent, despite seeing and naming some of their “flaws”. This way, children avoid “split-parent identification” [78]—solving the conflict of loyalty by unambiguously identifying with only one of the parents.

Ad (3) Unfortunately, the divorce and the conflict of adults also prompt their search for allies among children. There is a serious risk of differential treatment of children by their parents [21,22,23]. The adult chooses a child to approach, talks to them more often, treats them better, wants to “buy” the child’s feelings and thus starts to distinguish them from their siblings. The remaining children see the favoring of their “chosen” brother or sister. At the same time, the “chosen” child may experience a sense of rejection from the other parent who remains distant and perhaps has even already moved out of the house (when separating siblings from other children). In the second discussed case, the teenager had a feeling of his younger sister being favored by his mother and her partner. Already during the family crisis, he felt he was being treated worse by their mother compared to his sister, and this feeling intensified after the family reconstructed, and the boy’s experience was intensified by the attitude of his mother’s new partner. Remaining under the care of his father and his partner, he did not feel that his siblings (he and his half-sisters) were treated differently and unfairly.

The second aim of the presented report was to look at the possibility of using play as a diagnostic tool in the examination of a child in a situation of divorce. We presented the results of the diagnosis in which play was used as the key tool to assess the situation of children in families post-divorce. A frequent limitation of numerous previous studies on the impact of divorce on the functioning of children was the use of self-reports or observation methods that focus primarily on assessing the frequency of behaviors—for example, how often do siblings fight with each other? [99,100,101,102]. However, there is a difference between the visible behaviors and the feelings that siblings actually experience [103,104,105]. For example, when simply observing, we could positively evaluate the behavior of an older brother caring for a younger sister, while he could be motivated by the desire to control her actions and her relationship with a new member of the reconstructing family. Similarly, an observer can perceive a conflict between children as severe because sibling interactions often take the form of arguments or fights, while for children themselves, these situations are irrelevant or are a way of being together.

Likewise, parents judge their children as not caring for one another because they do not see direct interactions between them, while for the siblings or one of the children, the very possibility of being together (or next to each other) is important. The sensitivity of siblings to each other’s needs in families after divorce is also rarely studied. Play can therefore be used as a more sensitive tool in the cases when sibling relations and mutual support take more subtle forms, such as when the mere presence is of importance, or for example, spending time together in the absence of direct supporting communication [65,74].

Additionally, in many reports, only one perspective is used for relations among siblings, while research on family relations should take into account several of them. Those perspectives include: insider—the child evaluates their relationship with siblings; outsider—observer, researcher; participant observer—parent, who is not directly involved in child interactions [106]. Each perspective provides unique knowledge, and research conducted by Noller [32], among others, shows that between mothers and children, there are significant differences in perception of differential treatment of siblings by parents. Although siblings share experiences, each of them may experience changes in the family and relationships with each other differently [56]. Thus, only an analysis of similarities and inconsistencies in these assessments can provide a deeper insight into those relationships and a more reliable assessment of them.

## 6. Conclusions

Although we know a lot about the optimal parent–child relationship, also in the event of divorce, there is still a lack of knowledge about what determines “good” sibling relationships (lack of “sibling ideology”) [107]. In particular, the literature on the situation of siblings experiencing the separation of their parents and separation from their siblings after divorce as a result of random events or the decisions of adults is modest. Its review and analysis of the presented cases also help notice the complexity of the situation and the resulting difficulty for diagnosticians. For example, just the variety of sibling constellations—how many children there are, what their age is, who the eldest is, the sister or brother—determines this complexity and makes it necessary to consider each case very individually. In addition, the dynamics of sibling relationships before, during and after divorce have to be considered, and what will change if the sibling separation is maintained in the long term needs to be anticipated.

## Appendix A

1. “MONSTER” RIVER is a game used for diagnosing the strength of a child’s relationship with family members and a child’s assessment concerning resources for coping with difficult situations possessed by members of the family. The choices made by a child in the form of a game allow understanding of the emotional hierarchy of persons participating in the child’s life. The game includes a difficult situation, in a symbolic manner representing a crisis that a child experiences in the face of a family falling apart. By selecting soft toys representing family members, the child indicates the system’s integrity (who has been mentioned and selected), and by selecting the order in which the toys are transferred to the other side of a dangerous river, it points to the emotional hierarchy of persons participating in the child’s life;
2. WHO CAN HELP? is a game used for diagnosing the hierarchy within a family related to the psychological resources possessed by family members concerning coping with problems. The game allows understanding to what extent a child has a sense that it can take advantage of the parents as sources of support and what persons it addresses with its problems. The researcher presents a “problem”—a large item representing a difficult situation and says to the child: “This is a problem, your large problem. You know you won’t be able to deal with it on your own. Who will you ask for help?” In the “Who can help?” game, it is possible to gain information concerning who the child considers being a person taking care of its needs, especially by supporting it in difficult situations;
3. WHEELBARROW FOR PROBLEMS is used for diagnosing types of problems experienced by a child and allows for assessing their importance and hierarchy. A more valuable version of the game includes the possibility for a child to list problems, the manner and language used by the child to describe a problem allows to learn to what extent the child participates in the discussions of adults concerning a difficult situation. It is also possible to determine whether a child is protected by adults in a crisis situation—in such a case, the child’s worry appears at the top of the hierarchy of problems (“I don’t have a Barbie doll”) or the child worries about issues of adults (“We don’t have enough money”);
4. QUEUE TO THE HEART is a game used to assess the emotional relations of a child with significant persons and allows determining who the child has positive feelings for and what is the emotional hierarchy of people important in the child’s life. The game also allows assessing the number of people and the system they come from—whether only from the child’s family (parents and siblings) or are there also people from the family of origin (grandmother, grandfather, aunts, uncles, cousins), newly established families (step-father, stepmother, partners of the parents) or from outside the family (friends, teachers);
5. MEDALS is a game used so that the child can assess the parenting competencies of the mother and father, the degree of satisfying the child’s needs by each of the parents and also allowing to assess the strength of the child’s loyalty towards each of the parents. It allows understanding of how the child perceives both parents as caretakers and guardians, meaning persons taking care of it in various fields of life and upbringing. Due to ethical reasons, it is not correct to ask the child direct questions emphasizing its loyalty conflict (who does it better, mom or dad?) Awarding parents with medals unveils the ability to differentiate a parent’s competencies in terms of coping with various obligations and also allows the researcher to understand which of the parents, as perceived by the child, takes better care of the child’s needs;
6. TWO HOUSES is a game focused on understanding the care-upbringing competencies of parents as perceived by the child, as well as the child’s relations with the parents. The “Two houses” game can be useful, especially when both parents fight to have custody over the child and strive for the child to spend the maximum amount of time with the mother or father. In such a case, the child may prefer one home over the other or begins to cope with the parent’s splitting and lack of possibility to spend time with both parents in one place at one time, saying that it has “two houses.” The aim of the diagnosis is to determine which of the parents expresses better care-upbringing competencies, and during the assessment process, the perspective of the child and its sense of the degree of meeting its needs in each of the homes are also important. The child’s task is to assign various sentences concerning care and the child’s needs to one of the houses, meaning in which of the two houses a given need is satisfied;
7. PICTURE OF THE RELATIONSHIPS is used for diagnosing emotional relations between family members and conflicts between them as perceived by the child. It allows assessing the degree to which the child understands the mutual sympathies and dislikes between close and distant family members (the parents’ families of origin). Children usually understand mutual relations between members of a family in crisis better than adults may think.

## References

[1] Bryceson D., Vuorela U. (2020). The Transnational Family: New European Frontiers and Global Networks.

[2] Goldenberg H., Goldenberg I. (2012). Family Therapy: An Overview.

[3] Poland Divorce Rate 2020, Statista. https://www.statista.com/statistics/957237/poland-divorce-rate/.

[4] Cudak H. (2011). Dysfunkcje rodziny i jej zagrożenia opiekuńczo-wychowawcze. Pedagog. Rodz..

[5] Szredzińska R. (2017). Zdrowie dzieci i młodzieży. Dziecko Krzywdzone Teor. Bad. Prakt..

[6] Błażek M., Lewandowska-Walter A. (2017). Rozwód Jako Proces: Perspektywa Dorosłych i Dzieci.

[7] Kruk E. (2013). The Equal Parent Presumption: Social Justice in the Legal Determination of Parenting after Divorce.

[8] Kruk E. (2012). Arguments for unequal parental responsibility presumption in contested child custody. Am. J. Fam. Ther..

[9] Nielsen L. (2014). Shared physical custody: Summary of 40 studies on outcomes for children. J. Divorce Remarriage.

[10] Nielsen L. (2017). Re-examinig the Research on parental Conflict, Coparenting, and Custody Arrangments. Psychol. Public Policy Law.

[11] Garcia M.M., Shaw D.S., Winslow E.B., Yaggi K.E. (2000). Destructive sibling conflict and the development of conduct problems in young boys. Dev. Psychol..

[12] van Dijk R., van der Valk I.E., Buist K.L., Branje S., Deković M. (2022). Longitudinal associations between sibling relationship quality and child adjustment after divorce. J. Marriage Fam..

[13] Furstenberg F.F., Kiernan K.E. (2001). Delayed parental divorce: How much do children benefit?. J. Marriage Fam..

[14] Vélez C.E., Wolchik S.A., Tein J.Y., Sandler I. (2011). Protecting children from the consequences of divorce: A longitudinal study of the effects of parenting on children’s coping processes. Child Dev..

[15] Cummings E.M., Davies P.T. (2010). Marital Conflict and Children: An Emotional Security Perspective.

[16] Błażek M., Kaźmierczak M., Lewandowska-Walter A., Rostowska W.T., Jarmołowska A. (2010). Więzi uczuciowe i postawy wychowawcze w rodzinach o ograniczonych kompetencjach opiekuńczo-wychowawczych. Rozwojowe i Wychowawcze Aspekty Życia Rodzinnego.

[17] Poortman A.R., Voorpostel M. (2009). Parental divorce and sibling relationships: A research note. J. Fam. Issues.

[18] Rowen J., Emery R.E. (2019). Parental denigration reports across parent–child dyads: Divorced parents underreport denigration behaviors compared to children. J. Child Custody.

[19] United Nations International Children’s Emergency Fund (UNICEF) (2007). Implementation Handbook for the Convention on the Rights of the Child UNICEF.

[20] Prawo.pl. https://www.prawo.pl.

[21] Brody G., Stoneman Z., Brody G.H. (1996). A Risk-Amelioration Model of Sibling Relationships: Conceptual Underpinnings and Preliminary Findings. Sibling Relationships: Their Causes and Consequences.

[22] Dunn J., Smith P.K., Hart C.H. (2002). Sibling relationships. Blackwell Handbook of Childhood Social Development.

[23] Shanahan L., McHale S.M., Crouter A.C., Osgood D.W. (2008). Linkages between parents’ differential treatment, youth depressive symptoms, and sibling relationships. J. Marriage Fam..

[24] Dunn J., Hindle D., Sherwin-White S. (2014). Sibling relationships across the life-span. Sibling Matters: A Psychoanalytic, Developmental, and Systemic Approach.

[25] Boer F., Goedhart A.W., Treffers P.D. (1992). Siblings and their parents. Children’s Sibling Relationships: Developmental and Clinical Issues.

[26] Seginer R. (1998). Adolescents’ perceptions of relationships with older sibling in the context of other close relationships. J. Res. Adolesc..

[27] Volling B.L., Belsky J. (1992). The contribution of mother-child and father-child relationships to the quality of sibling interaction: A longitudinal study. Child Dev..

[28] Patterson G.R., Olweus D., Block J., RadkeYarrow M. (1986). The contribution of siblings to training for fighting: A microsocial analysis. Development of Antisocial and Prosocial Behavior: Research Theories and Issues.

[29] Bedford V.H., Volling B.L., Lang F.R., Fingerman K.L. (2004). A Dynamic Ecological Systems Perspective on Emotion Regulation Development within the Sibling Relationship Context. Growing Together: Personal Relationships Across the Lifespan.

[30] Criss M.M., Shaw D.S. (2005). Sibling relationships as contexts for delinquency training in low-income families. J. Fam. Psychol..

[31] Kim J.Y., McHale S.M., Wayne Osgood D., Crouter A.C. (2006). Longitudinal course and family correlates of sibling relationships from childhood through adolescence. Child Dev..

[32] Noller P. (2005). Sibling relationships in adolescence: Learning and growing together. Pers. Relatsh..

[33] Bretherton I., Munholland K.A., Cassidy J., Shaver P.R. (2008). Internal working models in attachment relationships: Elaborating a central construct in attachment theory. Handbook of Attachment: Theory, Research, and Clinical Applications.

[34] Kan M.L., McHale S.M., Crouter A.C. (2008). Interparental incongruence in differential treatment of adolescent siblings: Links with marital quality. J. Marriage Fam..

[35] Richmond M.K., Stocker C.M., Rienks S.L. (2005). Longitudinal associations between sibling relationship quality, parental differential treatment, and children’s adjustment. J. Fam. Psychol..

[36] Solmeyer A.R., Killoren S.E., McHale S.M., Updegraff K.A. (2011). Coparenting around siblings’ differential treatment in Mexican-origin families. J. Fam. Psychol..

[37] Jenkins J.M., Dunn J., O’Connor T.G., Rasbash J., Behnke P. (2005). Change in maternal perception of sibling negativity: Within- and between-family influences. J. Fam. Psychol..

[38] Kowal A., Kramer L., Krull J.L., Crick N.R. (2002). Children’s perceptions of the fairness of parental preferential treatment and their socioemotional well-being. J. Fam. Psychol..

[39] Dunn J. (2005). Commentary: Siblings in their families. J. Fam. Psychol..

[40] Campbell L.D., Connidis I.A., Davies L. (1999). Sibling ties in later life: A social network analysis. J. Fam. Issues.

[41] Milevsky A., Levitt M.J. (2005). Sibling support in early adolescence: Buffering and compensation across relationships. Eur. J. Dev. Psychol..

[42] Ponzetti J.J., James C.M. (1997). Loneliness and sibling relationships. J. Soc. Behav. Personal..

[43] Davies P.T., Parry L.Q., Bascoe S.M., Martin M.J., Cummings E.M. (2019). Children’s vulnerability to interparental conflict: The protective role of sibling relationship quality. Child Dev..

[44] Brody G.H., Stoneman Z., McCoy J.K. (1994). Forecasting sibling relationships in early adolescence from child temperaments and family processes in middle childhood. Child Dev..

[45] Hetherington E.M., Cowen P.A., Hetherington P.A. (2013). The role of individual differences and family relationships in children’s coping with divorce and remarriage. Family Transitions.

[46] Wallerstein J., Lewis J.M. (2007). Sibling outcomes and disparate parenting and stepparenting after divorce: Report from a 10-year longitudinal study. Psychoanal. Psychol..

[47] Miller G.H. (2002). Review of the book *The unexpected legacy of divorce: A 25 year landmark study*, by J. Wallerstein, J. Lewis & S. Blakeslee. J. Am. Acad. Child Adolesc. Psychiatry.

[48] Abbey C., Dallos R. (2004). The Experience of the Impact of Divorce on Sibling Relationships: A Qualitative Study. Clin. Child Psychol. Psychiatry.

[49] Noller P., Feeney J.A., Peterson C.C., Sheehan G., Socha T.J., Stamp G.H. (1995). Learning conflict patterns in the family: Links between marital, parental, and sibling relationships. Parents, Children, and Communication: Frontiers of Theory and Research.

[50] Brody G.H. (1998). Sibling relationship quality: Its causes and consequences. Annu. Rev. Psychol..

[51] Bank S., Kahn M.D., Lamb M.E., Sutton-Smith B., Sutton-Smith B., Lamb M.E. (2014). Intense sibling loyalties. Sibling Relationships Their Nature and Significance across the Lifespan.

[52] Jenkins J., Boer F., Dunn J. (1992). Sibling relationships in disharmonious homes: Potential difficulties and protective effects. Children’s Sibling Relationships: Developmental and Clinical Issues.

[53] Lanthier R.P., Furman W. Stress and sibling relationship quality in middle childhood. Proceedings of the Midwestern Psychological Association.

[54] Dunn J., Slomkowski C., Beardsall L. (1994). Sibling relationships from the preschool period through middle childhood and early adolescence. Dev. Psychol..

[55] Kunz J. (2001). Parental divorce and children’s interpersonal relationships: A meta-analysis. J. Divorce Remarriage.

[56] Smart C. (2006). Children’s narratives of post-divorce family life: From individual experience to an ethical disposition. Sociol. Rev..

[57] Pike A., Coldwell J., Dunn J.F. (2005). Sibling relationships in early/middle childhood: Links with individual adjustment. J. Fam. Psychol..

[58] Sheehan G., Darlington Y., Noller P., Feeney J. (2004). Children’s perceptions of their sibling relationships during parental separation and divorce. J. Divorce Remarriage.

[59] Noller P., Feeney J.A., Sheehan G., Darlington Y., Rogers C. (2008). Conflict in divorcing and continuously married families: A study of marital, parent-child and sibling relationships. J. Divorce Remarriage.

[60] McGuire S., McHale S.M., Updegraff K. (1996). Children’s perceptions of the sibling relationship in middle childhood: Connections within and between family relationships. Pers. Relat..

[61] Eno M.M. (1985). Sibling relationships in families of divorce. J. Psychother. Fam..

[62] Isaacs M. Children’s long term adjustment to divorce. Proceedings of the American Orthopsychiatric Association Annual Meeting.

[63] Jenkins J.M., Smith M.A. (1990). Factors protecting children living in disharmonious homes: Maternal reports. J. Am. Acad. Child Adolesc. Psychiatry.

[64] Coleman M., Fine M.A., Ganong L., Downs K.J., Pauk N. (2001). When you’re not the Brady Bunch: Identifying perceived conflicts and resolution strategies in stepfamilies. Pers. Relat..

[65] Bush J.E., Ehrenberg M.F. (2003). Young persons’ perspectives on the influence of family transitions on sibling relationships: A qualitative exploration. J. Divorce Remarriage.

[66] Byng-Hall J. (1995). Creating a secure family base: Some implications of attachment theory for family therapy. Fam. Process.

[67] Boszormenyi-Nagy I., Spark G. (1973). Invisible Loyalties: Reciprocity in Intergenerational Family Therapy.

[68] Hooper L.M. (2008). Defining and Understanding Parentification: Implications for All Counselors. Ala. Couns. Assoc. J..

[69] Jurkovic G.J. (1997). Lost Childhoods: The Plight of the Parentified Child.

[70] Hooper L.M., DeCoster J., White N., Voltz M.L. (2011). Characterizing the magnitude of the relation between self-reported childhood parentification and adult psychopathology: A meta-analysis. J. Clin. Psychol..

[71] Hooper L.M., Doehler K., Jankowski P.J., Tomek S.E. (2012). Patterns of self-reported alcohol use, depressive symptoms, and body mass index in a family sample: The buffering effects of parentification. Fam. J..

[72] Borchet J., Lewandowska-Walter A., Połomski P., Peplińska A., Hooper L.M. (2020). We are in this together: Retrospective parentification, sibling relationships, and self-esteem. J. Child Fam. Stud..

[73] Marquardt E. (2005). Between Two Worlds: The Inner Lives of Children of Divorce.

[74] Jacobs K., Sillars A. (2012). Sibling support during post-divorce adjustment: An idiographic analysis of support forms, functions, and relationship types. J. Fam. Commun..

[75] Jacobs L.R. (2021). Separating Siblings in the Divorce. http://www.dc4k.org.

[76] Margolin G., Christensen A., John R.S. (1996). The continuance and spillover of everyday tensions in distressed and nondistressed families. J. Fam. Psychol..

[77] Conger K.J., Stocker C., McGuire S. (2009). Sibling socialization: The effects of stressful life events and experiences. New Dir. Child Adolesc. Dev..

[78] Schachter F.F., Lamb M.E., Sutton-Smith B. (1982). Sibling deidentification and split-parent identification: A family tetrad. Sibling Relationships: Their Nature and Significance Across the Lifespan.

[79] Axline V.M. (1969). Play Therapy.

[80] Freud A. (1965). Normality and Pathology in Childhood: Assessment and Development.

[81] Klein M. (1984). Love, Guilt, and Reparation, and Other Works, 1921–1945.

[82] Knell S.M., O’Connor K.W., Braverman L.M. (1997). Cognitive-Behavioral Play Therapy. Play Therapy: Theory and Practice.

[83] Deacon S.A., Piercy F.P. (2001). Qualitative Methods in Family Evaluation: Creative Assess-ment Techniques. Am. J. Fam. Ther..

[84] Eaker B. (1986). Unlocking the Family Secret in Family Play Therapy. Soc. Work.

[85] Arad D. (2004). If Your Mother Were an Animal, What Animal Would She Be? Creating Play-Stories in Family Therapy: The Animal Attribution Story-telling Technique (AASTT). Family Process.

[86] Satir V. (2000). Rodzina. Tu Powstaje Człowiek.

[87] Minuchin S. (1984). Families and Family Therapy.

[88] Lewandowska-Walter A., Błażek M., Bruski W. (2013). Diagnoza Więzi Rodzinnych Dzieci i Młodzieży.

[89] Elrod L.H. (1985). Enforcing Child Support Using the Revised Uniform Reciprocal Enforcement of Support Act. Juv. Fam. Court. J..

[90] Nichols W.C. (1986). Sibling subsystem therapy in family system reorganization. J. Divorce.

[91] Smyth B.M., McIntosh J.E., Emery R.E., Howarth S.L.H., Drozd L., Saini M., Olesen N. (2016). Shared-time parenting: Evaluating the evidence of risks and benefits to children. Parenting Plan Evaluations: Applied Research for the Family Court.

[92] Kier C., Lewis C. (1998). Preschool sibling interaction in separated and married families: Are same-sex pairs or older sisters more sociable?. J. Child Psychol. Psychiatry Allied Discip..

[93] Milevsky A. (2004). Perceived parental marital satisfaction and divorce: Effects on sibling relations in emerging adults. J. Divorce Remarriage.

[94] Panish J.B., Stricker G. (2001). Parental marital conflict in childhood and influence on adult sibling relationships. J. Psychother. Indep. Pract..

[95] Riggio H.R. (2001). Relations between parental divorce and the quality of adult sibling relationships. J. Divorce Remarriage.

[96] MacKinnon C.E. (1989). An observational investigation of sibling interactions in married and divorced families. Dev. Psychol..

[97] Anderson E.R., Rice A.M. (1992). Sibling Relationships during Remarriage. Monogr. Soc. Res. Child Dev..

[98] Gass K., Jenkins J., Dunn J. (2007). Are sibling relationships protective? A longitudinal study. J. Child Psychol. Psychiatry.

[99] Deater-Deckard K., Dunn J., Lussier G. (2002). Sibling relationships and social-emotional adjustment in different family contexts. Soc. Dev..

[100] Kramer L., Kowal A.K. (2005). Sibling relationship quality from birth to adolescence: The enduring contributions of friends. J. Fam. Psychol..

[101] Scharf M., Shulman S., Avigad-Spitz L. (2005). Sibling Relationships in Emerging Adulthood and in Adolescence. J. Adolesc. Res..

[102] Yeh H.C., Lempers J.D. (2004). Perceived Sibling Relationships and Adolescent Development. J. Youth Adolesc..

[103] Newman J. (1994). Conflict and friendship in sibling relationships: A review. Child Study J..

[104] Harden B.J., Denmark N., Saul D. (2010). Understanding the needs of staff in Head Start programs: The characteristics, perceptions, and experiences of home visitors. Child. Youth Serv. Rev..

[105] Andreyko B., Subashkevych I. (2020). Psychological analysis of sociogram and biographical method for investigating parents of children with special educational needs. J. Educ. Cult. Soc..

[106] Ostrovska K., Grabovska S. (2020). Motivational readiness of children to school in nuclear and single parent families. J. Educ. Cult. Soc..

[107] Stoneman Z. (2005). Siblings of children with disabilities: Research themes. Ment. Retard..

